# Ferroptotic propagation: from single-cell execution to tissue-scale death programs

**DOI:** 10.1038/s41422-026-01283-z

**Published:** 2026-07-30

**Authors:** Daolin Tang, Rui Kang, Guido Kroemer

**Affiliations:** 1 Department of Surgery, UT Southwestern Medical Center, Dallas, TX USA; 2 Université Paris Cité, Sorbonne Université, Inserm, Centre de Recherche des Cordeliers, Paris, France Centre de Recherche des Cordeliers, Equipe labellisée par la Ligue contre le cancer, Paris, France; 3 Institut Gustave Roussy, Université Paris-Saclay, INSERM US23/CNRS UAR 3655, Metabolomics and Cell Biology Platforms, UMS AMMICa, Villejuif, France; 4 Department of Biology, Hôpital Européen Georges Pompidou, AP-HP, Institut du Cancer Paris CARPEM, Paris, France

**Keywords:** Cell death, Cell signalling

## Abstract

Ferroptosis has long been regarded as a cell-autonomous form of regulated cell death driven by iron-dependent lipid peroxidation. Recent work, however, suggests that ferroptotic commitment can extend beyond the initiating cell, spreading to neighboring cells and, in some contexts, across tissue-scale distances. Multiple, non-mutually exclusive modes of contagion have now been described, including reactive oxygen species-triggered death waves, direct membrane contact-dependent transfer, and extracellular vesicle-mediated paracrine signaling. These findings redefine ferroptosis from a single-cell execution pathway into a spatially coordinated, multicellular process. In this perspective, we integrate mechanistic insights with experimental evidence on ferroptotic contagion and propose a unifying, multiscale framework in which distinct modes of transmission operate over different spatial scales, from short-range membrane transfer to longer-range oxidative and extracellular relay mechanisms. We discuss how nonlinear redox amplification, membrane biophysics, and tissue architecture together determine the dynamics, limits, and patterning of ferroptotic injury in vivo. This emerging framework has important implications for developmental tissue remodeling, the progression of organ injury, and ferroptosis-based cancer therapy. More broadly, defining how ferroptotic contagion is initiated, constrained, and manipulated therapeutically may help establish ferroptosis as a fundamental organizing principle for understanding tissue-level regulation and pathological escalation.

## Introduction

Cell death is executed by distinct molecular machineries and, in multicellular systems, rarely occurs in isolation.^1–3^ Across development, tissue homeostasis, and disease, cell death often emerges in spatially clustered, temporally coordinated, or even wave-like patterns.^4^ Similar transmission-like phenomena have been documented in other biological contexts, including apoptosis-induced apoptosis during developmental remodeling and the radiation-induced bystander effect, in which stressed or dying cells transmit pro-death signals to neighboring non-targeted cells through secreted factors or direct intercellular communication.^5,6^ These precedents illustrate a broader principle: cell death can unfold as a tissue-level process in which the demise of one cell actively shapes and sometimes determines the fate of its neighbors.

This conceptual framework is particularly relevant to ferroptosis, whose execution centers on membrane phospholipid peroxidation and is sustained by a chemically self-amplifying radical chain reaction.^7,8^ For many years, ferroptosis has been studied primarily as a cell-autonomous form of regulated cell death that occurs when iron-dependent lipid peroxidation overwhelms endogenous antioxidant defenses.^9^ Accordingly, much of the field has focused on intracellular determinants of susceptibility, including cellular iron handling, glutathione metabolism, the remodeling of polyunsaturated fatty acid (PUFA)-containing phospholipids, and both glutathione peroxidase 4 (GPX4)-dependent and GPX4-independent antioxidant defense pathways.^10,11^ This body of work has been instrumental in defining how individual cells cross the ferroptotic threshold, although it is increasingly evident that this threshold varies widely across cell types, metabolic states, and tissue contexts.^12–14^ Such variability implies the existence of spatial interactions capable of amplifying or buffering ferroptotic injury.

Yet this classical view does not fully explain the way ferroptotic injury often presents in tissues. In multiple pathological settings, affected cells are not distributed randomly, but instead form contiguous zones of degeneration, synchronized tubular epithelial loss, aligned myofiber injury, or expanding fronts of tissue damage, patterns that are difficult to reconcile with strictly cell-autonomous execution. Early conceptual work had already raised the possibility that ferroptosis might possess a collective property distinguishing it from other regulated necrotic programs, namely, the ability to spread across cell populations in space and time.^15^

Recent experimental studies now lend strong support to this idea (Table 1). In confluent cell monolayers, including epithelial models such as MCF10A mammary epithelial cells, ferroptotic death can propagate in wave-like spatiotemporal fronts through neighboring cells.^16,17^ In other systems, transmission depends on direct membrane contact with neighboring cells.^18^ In kidney injury, extracellular vesicles and secreted lipid mediators are sufficient to synchronize ferroptotic death across spatially contiguous tubular epithelial cells, thereby contributing to a self-amplifying “wave of tubular death” during the transition from acute kidney injury to chronic kidney disease.^16,19–21^

**Table 1:** Representative evidence supporting ferroptotic propagation across biological systems

Biological system	Propagation mode	Spatial range	Key mediators	In vivo evidence	Ref
Confluent epithelial monolayer	ROS trigger wave	Long-range (>5 mm)	ROS, iron	No	^22^
Epithelial monolayer	Membrane contact	Short-range	Membrane proximity	No	^18^
Kidney tubule	Extracellular vesicles	Segmental	WAC-AS1, EV RNA	Yes	^16,19^
Kidney graft	Metabolic relay	Tubular axis	Lactate, AARS1	Yes	^61^
Heart	Fiber-aligned spread	Tissue-level	Not fully defined	Yes	^94^
Embryonic limb	Trigger wave	Millimeter-scale	ROS	Yes	^22^
Cyanobacterial colony (*Microcystis*)	Lipid peroxide relay	Population-level	Truncated phospholipids	Yes	^90^

**Fig. 1:**
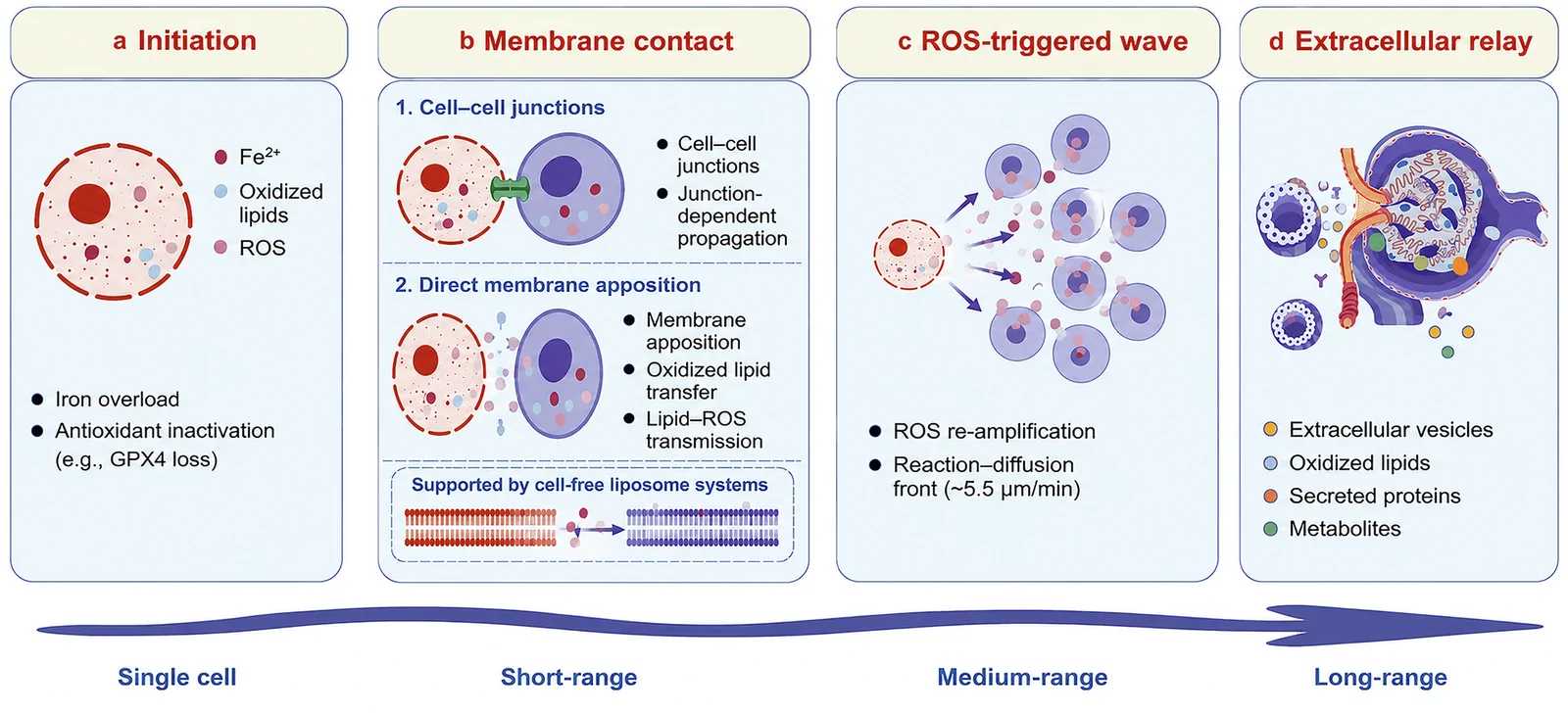
Conceptual framework of multiscale ferroptotic propagation. Ferroptotic spread may occur across multiple spatial scales through distinct but potentially interconnected mechanisms. **a** Initiation. Ferroptosis is first triggered at the single-cell level by intracellular iron overload and antioxidant inactivation, exemplified by GPX4 loss, leading to the accumulation of lipid peroxides and membrane damage. **b** Membrane contact. At short range, ferroptotic signals may propagate through cell–cell junctions, direct membrane apposition, and membrane-to-membrane transfer of oxidized lipids or lipid-derived ROS. Cell-free liposome models support the possibility that oxidized membrane states can be transmitted between closely apposed lipid bilayers. **c** ROS-triggered wave. At the tissue level, ferroptotic injury may spread as a medium-range reaction — diffusion front driven by ROS re-amplification, allowing local oxidative stress to propagate across neighboring cell layers. **d** Extracellular relay. Over longer range, extracellular mediators, including extracellular vesicles (EVs), oxidized lipids, secreted proteins, and metabolites, may relay ferroptotic signals through the extracellular microenvironment and extend the spatial boundaries of propagation. The bottom arrow illustrates the progression from single-cell initiation to short-range, medium-range, and long-range propagation, highlighting the multiscale nature of ferroptotic spread. This figure was created with BioGDP (https://biogdp.com/).

Taken together, these findings suggest that ferroptosis is better understood not only as a molecular death pathway but also as a spatially coordinated, multicellular process (Fig. 1). This perspective discusses the mechanisms that enable ferroptotic contagion across tissues and considers how this emerging concept reframes our understanding of development, disease, and therapeutic intervention.

## Defining ferroptotic propagation

One of the central conceptual challenges in this field lies in distinguishing true propagation from coincidental co-injury. Spatial clustering of dead cells, even when highly ordered, does not by itself demonstrate that one dying cell actively drives the death of another. Cells occupying the same microenvironment may simply be exposed to a shared upstream insult, such as hypoxic, ischemic, toxic, or inflammatory stress, leading to simultaneous but independent ferroptotic responses.

For this reason, the term ferroptotic propagation should be reserved for situations in which an initiating ferroptotic event causally induces secondary ferroptotic death in neighboring or spatially connected cells through a transmissible mechanism. In our view, at least three operational criteria should be considered: spatiotemporal ordering, mechanistic continuity, and ferroptosis specificity. These criteria collectively distinguish coordinated tissue dynamics from coincidental injury.

An important prerequisite for evaluating these criteria is the presence of a spatially defined initiation site. Without a spatially restricted and experimentally identifiable origin of ferroptotic stress, ordered patterns of cell death could simply reflect pre-existing gradients in ferroptosis susceptibility within heterogeneous microenvironments rather than true directional propagation. Therefore, controlled initiation is essential for interpreting spatiotemporal ordering as evidence of causal spread.

First, secondary death should occur in a spatially and temporally ordered manner that is consistent with directional spread rather than random coincidence. For example, in highly confluent epithelial monolayers, erastin-induced ferroptosis does not arise stochastically throughout the culture.^22^ Instead, cells undergo progressive swelling and death in a wave-like front that advances across the monolayer over time.^22^ This ordered progression, initiated from a defined focus of ferroptotic stress, strongly supports a propagative process rather than simultaneous co-injury, implying directional information flow across a cell population.

Second, the secondary death event should remain mechanistically linked to the initiating signal. In renal injury models, extracellular vesicles isolated from hypoxia-treated human proximal tubular epithelial cells induce ferroptosis in previously untreated recipient tubular cells.^16^ The preservation of ferroptosis sensitivity in recipient cells provides evidence for mechanistic continuity, indicating that the transmitted signal retains functional fidelity rather than merely triggering nonspecific stress responses.

Third, the propagated event should retain ferroptosis-specific features. This distinction is particularly important because local tissue injury often generates generic inflammatory or oxidative bystander effects that may lead to nonspecific cell death. To qualify as ferroptotic propagation, secondary death should remain associated with canonical hallmarks such as lipid peroxide accumulation, iron dependence, and sensitivity to ferroptosis inhibitors. These features anchor the phenomenon within the ferroptotic regulatory network.^7,12,23^

At the same time, not all propagation-like phenomena are mechanistically equivalent. In tumor systems, for example, galectin-13 (LGALS13) released by ferroptotic cells does not appear to directly convey oxidative species to neighboring cells. Instead, it lowers the ferroptotic threshold of recipient cells by destabilizing the membrane localization of solute carrier family 7 member 11 (SLC7A11), the light-chain subunit of system xc^–^, responsible for cystine uptake, through CD44-dependent signaling.^24^ This raises a conceptual distinction between active signal transmission, in which a death-inducing signal is directly relayed, and state transmission, in which recipient cells are rendered permissive to subsequent ferroptosis. Whether both processes should be considered bona fide propagation remains an open conceptual question and clearly represents a key definitional boundary for the field.

## Why ferroptosis is inherently propagative

Ferroptosis is particularly suited to propagation because the chemistry underlying its execution is intrinsically self-amplifying.^25^ Unlike apoptosis, in which caspase activation remains largely confined within individual cells,^26^ ferroptosis is driven by the autoxidation of polyunsaturated phospholipids within cellular membranes.^27^ Once initiated, lipid alkoxyl and peroxyl radicals continuously abstract hydrogen atoms from neighboring phospholipid acyl chains, generating new radical intermediates and sustaining a chain reaction that can, at least in principle, extend across membrane domains.^28^ Foundational studies of lipid autoxidation chemistry have been essential in establishing this framework and in showing that radical-trapping antioxidants act by terminating the chain-propagation phase of lipid peroxidation,^29,30^ thereby defining the kinetic control points that govern ferroptotic escalation.^31,32^

This chemistry becomes biologically meaningful because the substrate is the membrane itself. Unlike signaling pathways restricted to cytosolic proteins, the lipid bilayer forms a continuous, laterally diffusive structure in which oxidized lipids and radical intermediates can, at least in principle, spread within and between membrane microdomains.^33^ Consistent with this concept, in vivo zebrafish injury models have demonstrated that lipid peroxidation can act as a spatial redox relay over hundreds of micrometers, providing a biologically relevant framework for how oxidative signals may propagate across multicellular tissues.^34^ Such chemistry is likely to be relevant in dense epithelial sheets, aligned muscle fibers, and compact tumor nests, where adjacent plasma membranes lie in close proximity and often share similar lipid composition, iron availability, and redox conditions.

Support for this idea comes not only from cell-based observations but also from simplified biophysical systems. In a minimal cell-free liposome system, iron-dependent lipid peroxidation was reconstituted between directly apposed membranes, indicating that membrane proximity alone may be sufficient to sustain oxidative spread.^18^ This observation shifts the discussion beyond descriptive cell biology and suggests that ferroptotic propagation may be encoded, at least in part, in the physicochemical properties of oxidized lipid bilayers rather than in cell-specific signaling programs.

At the same time, this intrinsic capacity for spread should not be mistaken for inevitable propagation in vivo. Whether a local ferroptotic event expands into surrounding tissue remains strongly constrained by threshold behavior. Redox-buffering systems, including GPX4,^35,36^ AIF family member 2 (AIFM2, also known as ferroptosis suppressor protein 1 (FSP1)),^37–41^ and endogenous radical-trapping molecules such as glutathione,^35,42,43^ 7-dehydrocholesterol,^44–46^ and hydropersulfides,^47–49^ together with membrane damage repair machinery such as the endosomal sorting complex required for transport III (ESCRT-III),^50,51^ do not operate as simple on-off switches. Instead, they continuously buffer cells against entering a runaway peroxidative state and help preserve membrane integrity under sublethal oxidative stress. Spatial spread is therefore more likely to occur only when the local oxidative burden exceeds the combined redox-buffering and membrane repair capacity of neighboring cells. This tipping point may convert localized damage into expanding tissue injury.

Collectively, these features indicate that ferroptosis is intrinsically predisposed to propagation because its execution chemistry, membrane-based substrate, and threshold-dependent defense systems all favor nonlinear amplification. Such a system-level property may link molecular reactions to tissue-scale dynamics.

## Reactive oxygen species (ROS)-triggered wave propagation

The most compelling evidence for tissue-scale ferroptotic propagation to date comes from a trigger-wave model established in highly confluent epithelial monolayers, in which localized ferroptotic stress gives rise to self-sustaining oxidative fronts that travel across the culture over distances exceeding 5 mm at a nearly constant velocity of ~5.5 μm/min.^22^ The quantitative features of this process are particularly informative. If long-range spread were driven solely by passive diffusion of ROS, one would expect progressive attenuation of signal strength and a corresponding decline in propagation speed with increasing distance. Instead, the maintenance of an approximately constant wave velocity strongly supports the existence of a reaction-diffusion-like excitable system, in which oxidative stress in one region triggers de novo ROS amplification in adjacent cells, thereby enabling a self-propagating wave of ferroptotic contagion.

Mechanistically, this wave behavior depends on continuous local re-amplification rather than simple ROS transfer. Propagation is attenuated by iron chelation and accelerated by iron supplementation, consistent with a critical role for iron-dependent Fenton chemistry.^22^ In addition, inhibition of nicotinamide adenine dinucleotide phosphate (NADPH) oxidase activity and perturbation of glutathione synthesis significantly impair wave progression, further indicating that recipient cells must actively engage their own redox machinery for the front to advance.^22^ In this framework, ROS function less as long-range diffusible killers than as local triggers that convert neighboring cells into secondary nodes of oxidative amplification.^52^

An implication of this model is that ferroptotic contagion may, under certain conditions, behave analogously to other excitable biological wave systems, such as calcium waves or spreading depolarization, in which local activation is continuously regenerated rather than physically transmitted over long distances.^53,54^ This distinction is conceptually important because it shifts the emphasis from ROS diffusion to self-propagating redox state transitions. Such a systems-level perspective aligns ferroptosis with canonical excitable media.

Particularly intriguing is the observation that comparable wave-like ferroptotic activity has also been identified during avian embryonic limb muscle remodeling, where oxidative fronts appear to contribute to the physiological elimination of transient muscle populations.^22^ This raises the possibility that ferroptotic propagation is not exclusively a pathological consequence of tissue injury but may also participate in developmental tissue patterning and remodeling during embryogenesis.

At the same time, the broader applicability of this model remains unresolved. Most mechanistic dissection has thus far been performed in highly confluent two-dimensional cell culture systems that maximize cell–cell coupling and minimize extracellular dilution. Whether similar trigger-wave dynamics can be sustained in complex three-dimensional tissues, where geometry, extracellular matrix, and heterogeneous antioxidant buffering may substantially alter propagation behavior, remains an important open question and a critical test of the model’s physiological generalizability.

## Membrane contact-dependent propagation

A mechanistically distinct mode of ferroptotic spread has recently emerged from studies focusing on direct membrane contact rather than long-range oxidative signaling. In a confluent epithelial monolayer system, spatially restricted ferroptosis was induced using an optogenetic GPX4-degradation platform, allowing localized initiation of ferroptotic stress with high spatial precision.^18^ Under these conditions, secondary death was not observed as a broad oxidative front. Instead, ferroptotic contagion was largely confined to cells positioned immediately adjacent to the initiating focus, indicating that propagation operated over a much shorter spatial scale, consistent with a contact-limited transmission mechanism.

Several observations from this system strongly support a contact-dependent mechanism. First, increasing the physical distance between neighboring cells sharply reduced the frequency of secondary death, suggesting that close membrane apposition is a critical determinant of spread.^18^ Second, disruption of α-catenin-dependent cell–cell adhesion, which destabilizes adherens junctions and increases intercellular spacing, almost completely abolished propagation.^18^ These findings argue against a purely diffusible extracellular mediator and instead point toward a mechanism that depends on direct membrane proximity.

Perhaps the most compelling evidence comes from reductionist reconstitution experiments. In a cell-free liposome–liposome contact system, iron-dependent lipid peroxidation could be transmitted between directly apposed bilayers in the absence of cytosolic signaling components, indicating that the oxidized membrane state itself can, under appropriate conditions, propagate across closely juxtaposed membranes.^18^ This observation suggests that ferroptotic spread may, at least in part, be encoded in the physicochemical properties of lipid bilayers rather than relying exclusively on canonical intracellular signaling pathways.^55^

These findings should also be interpreted in the context of cell density, as ferroptotic susceptibility itself is known to be strongly modulated by confluency and cell–cell contact.^56,57^ High-density epithelial states can alter redox sensitivity, junctional organization, and membrane apposition, all of which have previously been shown to inhibit ferroptosis.^56,57^ Accordingly, whether contact-dependent spread reflects a fundamentally distinct transmission mechanism or instead emerges, in part, from density-conditioned membrane architecture remains an important consideration.

At first glance, this contact-dependent model appears to stand in tension with the long-range ROS trigger-wave mechanism discussed above. However, these models are not necessarily mutually exclusive. Rather, they are more likely to represent distinct density- and distance-dependent spatial regimes of ferroptotic propagation. Long-range spread may be dominated by redox excitability and local ROS re-amplification, whereas short-range propagation may be governed primarily by membrane biophysics, intercellular spacing, and junctional architecture, which together define a continuum of spatial coupling mechanisms.

An important unresolved question is whether these two modes coexist within the same tissue. In densely packed epithelia, for example, direct membrane transfer may dominate early local spread, while trigger-wave dynamics may account for subsequent expansion across larger tissue territories. Resolving the relative contributions of these mechanisms in vivo remains an important challenge for the field and will likely require quantitative imaging approaches capable of linking molecular events to tissue-scale dynamics.

## Extracellular relay and paracrine propagation

Although direct membrane contacts and ROS-triggered waves provide two compelling frameworks for ferroptotic spread, neither fully explains how ferroptosis extends across anatomically separated yet functionally coupled cells. This issue is particularly relevant in epithelial organs such as the kidney, where tubular cells are aligned along the nephron axis but are not necessarily in continuous plasma membrane contact.^16,19,20^ This spatial arrangement demands relay mechanisms capable of bridging physical discontinuities.

The strongest evidence for this mode of propagation comes from renal injury models. In hypoxia-treated human proximal tubular epithelial cells, the release of apical small extracellular vesicles from the luminal surface is markedly increased.^16^ Transfer of these vesicles to previously untreated recipient tubular cells induces a canonical ferroptotic phenotype, including loss of GPX4, accumulation of lipid ROS, increased malondialdehyde levels, and progressive membrane damage. This effect is attenuated by both ferrostatin-1 and RNase pretreatment, indicating that the propagated signal depends on biologically active RNA cargo rather than passive membrane fragments or nonspecific debris.^16^

This mechanism has been further strengthened by in vivo evidence from murine bilateral renal ischemia-reperfusion injury models, in which vesicles released from injured tubules have been shown to carry the long non-coding RNA WAC-AS1 and suppress antioxidant defenses in neighboring tubular cells.^19^ Mechanistically, this vesicular cargo acts through the glutamine-fructose-6-phosphate transaminase 1 (GFPT1)-hexosamine biosynthesis pathway and transcriptional regulator BTB and CNC homolog 2 (BACH2)-dependent repression of the SLC7A11-GPX4 axis, thereby lowering the ferroptotic threshold of adjacent cells.^19^ Particularly compelling is the observation that pharmacological inhibition of vesicle biogenesis with GW4869 restricts the spatial expansion of tubular injury, supporting a causal role for extracellular vesicle-mediated propagation in vivo.^19^

At the same time, extracellular vesicles do not function exclusively as pro-propagation signals. Their effects appear to be highly context-dependent. For example, prominin-2 (PROM2)-mediated exosomes can promote ferroptosis resistance by exporting ferritin-containing multivesicular bodies and facilitating iron efflux, thereby reducing the intracellular labile iron pool.^58^ Similarly, vesicles generated under erastin-induced conditions, but not those released following RSL3 treatment, appear to dock more efficiently onto neighboring cells and accelerate secondary cell death.^59^ These findings suggest that vesicle-mediated signaling may either amplify or restrain ferroptosis depending on cargo composition and uptake efficiency, highlighting extracellular vesicles as bidirectional regulators of ferroptotic susceptibility rather than unidirectional death signals. Notably, RSL3 is widely used as a pharmacological inhibitor of GPX4, although studies in cell-free systems suggest that it may also exhibit inhibitory activity toward thioredoxin reductase 1 (TXNRD1), a key NADPH-dependent antioxidant enzyme that maintains thioredoxin-mediated redox homeostasis.^60^

A further layer of extracellular relay may be mediated by diffusible metabolic signals. In graft kidney injury models, lactate released from ischemia-reperfusion-injured tubular cells can transmit a ferroptosis-permissive state to neighboring cells through metabolic shuttling.^61^ Extracellular lactate induces alanyl-tRNA synthetase 1 (AARS1)-dependent lactylation of nucleophosmin 1 (NPM1), leading to repression of SLC7A11 and impaired cystine metabolism, thereby facilitating ferroptotic trigger waves along tubular compartments.^61^ Conversely, extracellular metabolites may also restrain propagation by altering membrane lipid composition and reducing ferroptosis susceptibility. For example, exogenous monounsaturated fatty acids (MUFAs) can suppress ferroptotic sensitivity by remodeling membrane phospholipid composition and limiting lipid peroxidation.^62^ Measurement of the PUFA-to-MUFA ratio may help predict ferroptosis sensitivity,^63–65^ underscoring the role of metabolic context as a determinant of ferroptotic transmissibility.

Beyond vesicles, secreted proteins and oxidized lipid mediators may also act as paracrine amplifiers of ferroptotic susceptibility. In tumor and myocardial ischemia-reperfusion injury models, extracellular 4-hydroxynonenal (4-HNE), a lipid peroxidation-derived aldehyde, enhances ferroptotic susceptibility in neighboring cells.^66,67^ In addition, ferroptotic cancer cells secrete extracellular factors, including LGALS13, that modulate the antioxidant state of adjacent cells, thereby priming them for subsequent ferroptotic collapse.^24^ In kidney injury, platelet-activating factor (PAF) and PAF-like phospholipids are extracellular mediators of synchronized tubular ferroptosis.^68^ These oxidized lipid species destabilize membrane integrity and facilitate coordinated cell death along connected nephron segments, whereas platelet-activating factor acetylhydrolase 2 (PAFAH2) acts as an endogenous brake by detoxifying these relay signals and thereby restricting ferroptotic spread.^68^ This finding further supports the idea that oxidized lipid species themselves can function as bioactive relay signals capable of coordinating tissue-scale injury.

An additional consideration is the selective release of proteins under ferroptotic stress. Ferroptotic cells can release specific proteins, including GPX4, which has been implicated in limiting the immunogenicity of ferroptotic death through interaction with the zona pellucida glycoprotein 3 (ZP3) receptor on surrounding dendritic cells.^69^ Conversely, the extracellular release of decorin (DCN, an extracellular matrix-derived damage-associated signaling proteoglycan),^70^ high mobility group box 1 (HMGB1, an architectural DNA-binding factor in the cell nucleus),^71^ and nuclear receptor coactivator 4 (NCOA4, a mediator of ferritinophagy)^72,73^ may function as damage-associated alarm signals that promote inflammatory responses in the local microenvironment.^74–76^ Similarly, extracellular sequestosome 1 (SQSTM1, an autophagy receptor)^77^ has been shown to enhance ferroptotic susceptibility by upregulating acyl-CoA synthetase long-chain family member 4 (ACSL4), thereby promoting pancreatic necrosis.^78^ Whether such extracellular proteins also directly influence ferroptotic propagation (for example, by scavenging oxidized lipids, modulating iron handling, or reshaping the local redox landscape) remains an important open question.^79^

Rather than depending on a single relay mechanism, extracellular ferroptotic propagation is likely mediated by a context-dependent signaling network whose dominant components vary with tissue architecture and disease state. However, the precise molecular identity of the principal relay species remains unresolved. Although extracellular vesicles, oxidized phospholipids, secreted proteins, and lipid-derived electrophilic metabolites all represent plausible candidates, it remains unclear which mediators possess sufficient stability and diffusivity to support propagation across anatomically separated yet functionally connected cells. Whether any of these species can sustain long-range propagation beyond local paracrine signaling remains a major unresolved question for the field and a central target for future mechanistic and quantitative investigation.

## Threshold determinants: iron, lipids, redox buffering, and tissue architecture

**Fig. 2:**
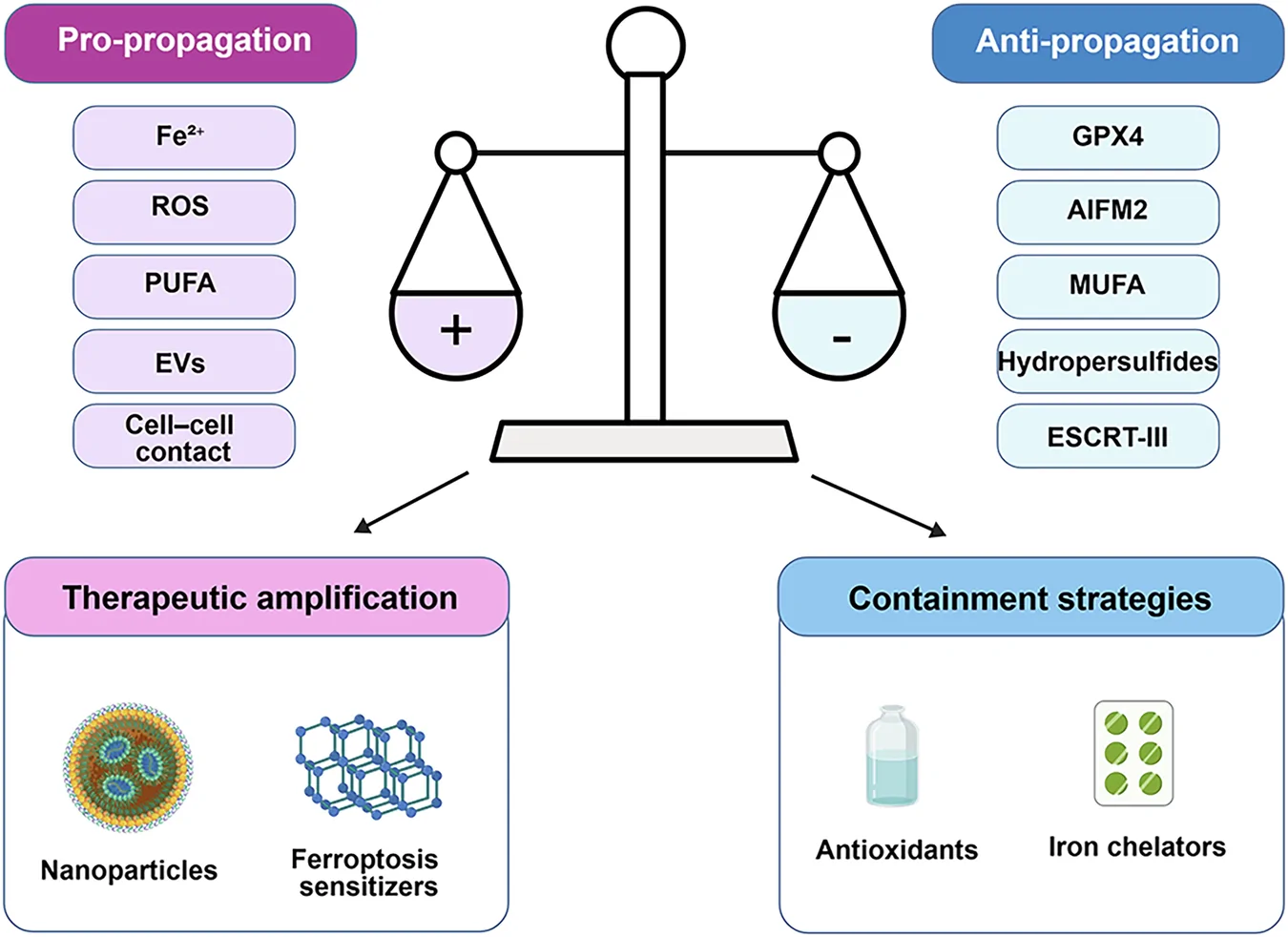
Determinants of the ferroptotic propagation threshold and translational modulation strategies. The spatial spread of ferroptosis is governed by the balance between propagation-promoting and propagation-restricting factors that together define a context-dependent propagation threshold. Pro-propagation determinants include labile iron (Fe^2+^), polyunsaturated fatty acid (PUFA)-enriched membranes, ROS amplification, extracellular vesicle (EV)-mediated signal relay, and cell–cell contact, all of which lower this threshold. In contrast, anti-propagation determinants include antioxidant defense systems such as GPX4 and AIFM2, hydropersulfide-mediated redox buffering, membrane stabilization by monounsaturated fatty acids (MUFA), and ESCRT-III-mediated repair, which collectively increase resistance to propagation. The lower panels illustrate translational strategies to modulate this threshold: therapeutic amplification to promote ferroptotic spread in cancer and containment strategies to restrict propagation in degenerative or inflammatory diseases using antioxidants and iron chelators. This figure was created with BioGDP (https://biogdp.com/).

A key conceptual advance in this field is the recognition that ferroptotic propagation is determined not only by the initiating signal but also by the susceptibility landscape of the recipient tissue. In many settings, the contagion of cellular demise depends as much on the local threshold for oxidative amplification as on the nature of the initial trigger, marking a shift from trigger-centric to susceptibility-centric thinking (Fig. 2).

Among these factors, iron availability is likely the most direct determinant.^80^ In trigger-wave models, extracellular iron supplementation significantly increases wave velocity, whereas iron chelation with deferoxamine markedly slows, and in some cases halts, propagation.^22^ Consistent with this notion, recent studies have identified the polyamine spermine as an endogenous iron chelator that restrains ferroptosis and confers protection in both cancer and tissue injury settings.^81^ A similar dependence has been observed in membrane contact-dependent systems, where extracellular iron chelation largely abolishes short-range spread between adjacent cells.^18^ These findings suggest that iron acts not simply as an initiator of ferroptosis but as a local amplification factor, enabling recipient cells to regenerate lipid peroxidation once exposed to an initiating oxidative cue, thereby functioning as a catalytic driver of propagation dynamics.

Membrane lipid composition represents a second major threshold determinant.^82–84^ This is particularly evident in triple-negative breast cancer models, where inhibition of diacylglycerol O-acyltransferase (DGAT)-dependent lipid droplet biogenesis redistributes PUFA species from neutral lipid storage pools into membrane phospholipids.^85^ The resulting increase in membrane unsaturation markedly enhances cellular susceptibility to lipid peroxidation and ferroptotic injury, thereby lowering the threshold for propagation once oxidative signals are initiated. In this context, recipient membranes are not passive targets; rather, their biophysical composition directly shapes whether incoming oxidative stress can be sustained and locally amplified.

Redox-buffering capacity introduces an additional layer of heterogeneity. In renal ischemia-reperfusion injury models, female mice display greater resistance to ferroptotic spread during acute kidney injury, with propagation along renal tubules largely absent compared with male mice.^21^ This protective phenotype has been linked to estradiol-dependent enhancement of endogenous radical-trapping capacity, including hydroxylated estrogen metabolites and increased glutathione hydropersulfide buffering.^21^ Biochemical studies further support this mechanism by showing that endogenous hydropersulfides act as potent chain-breaking antioxidants that directly quench lipid ROS and suppress the propagation step of lipid peroxidation.^86^ These findings suggest that organism-level physiological states, including hormonal milieu, metabolic status, and systemic redox balance, can act as intrinsic determinants of the spatial boundaries and propagation threshold of ferroptotic injury.

Tissue architecture imposes an equally important spatial constraint. In kidney injury models, ferroptotic spread frequently extends longitudinally along the tubular axis and appears largely confined within individual nephron segments.^19^ This anisotropic pattern suggests that geometry, polarity, and compartmentalization function as physical boundaries that shape the directionality of spread, effectively channeling ferroptotic injury along predefined anatomical paths. Similar principles are likely to apply in other highly organized tissues, where aligned fibers, epithelial junctions, or stromal barriers may channel or restrict ferroptotic expansion.

At the same time, the extracellular microenvironment is also likely to influence the spatial range of ferroptotic spread. Components of the extracellular matrix, the basement membrane, and interstitial metabolites may act as diffusion barriers or local redox buffers that restrict the movement of oxidized lipids, iron species, and paracrine death signals. In this context, propagation should be considered a function not only of intracellular susceptibility but also of extracellular containment capacity, establishing a balance between amplification and confinement. Recent work further supports this concept by showing that tumor acidosis-driven glycocalyx remodeling restricts extracellular lipid scavenging and suppresses ferroptosis, thereby shaping ferroptotic susceptibility and the potential spatial boundaries of propagation.^87^ A related principle may also operate in inflammatory tissue niches, where oxidized lipids, often associated with lipoprotein particles such as low-density lipoprotein (LDL), are cleared by resident immune cells, thereby modulating local ferroptotic thresholds and potentially influencing the permissiveness of propagation.^88^

More broadly, this highlights an underappreciated principle in the field: ferroptotic propagation is shaped not only by intracellular biochemistry but also by tissue-scale geometry and extracellular spatial constraints, linking molecular reactions to anatomical structure.

## Execution heterogeneity and collective death dynamics

**Fig. 3:**
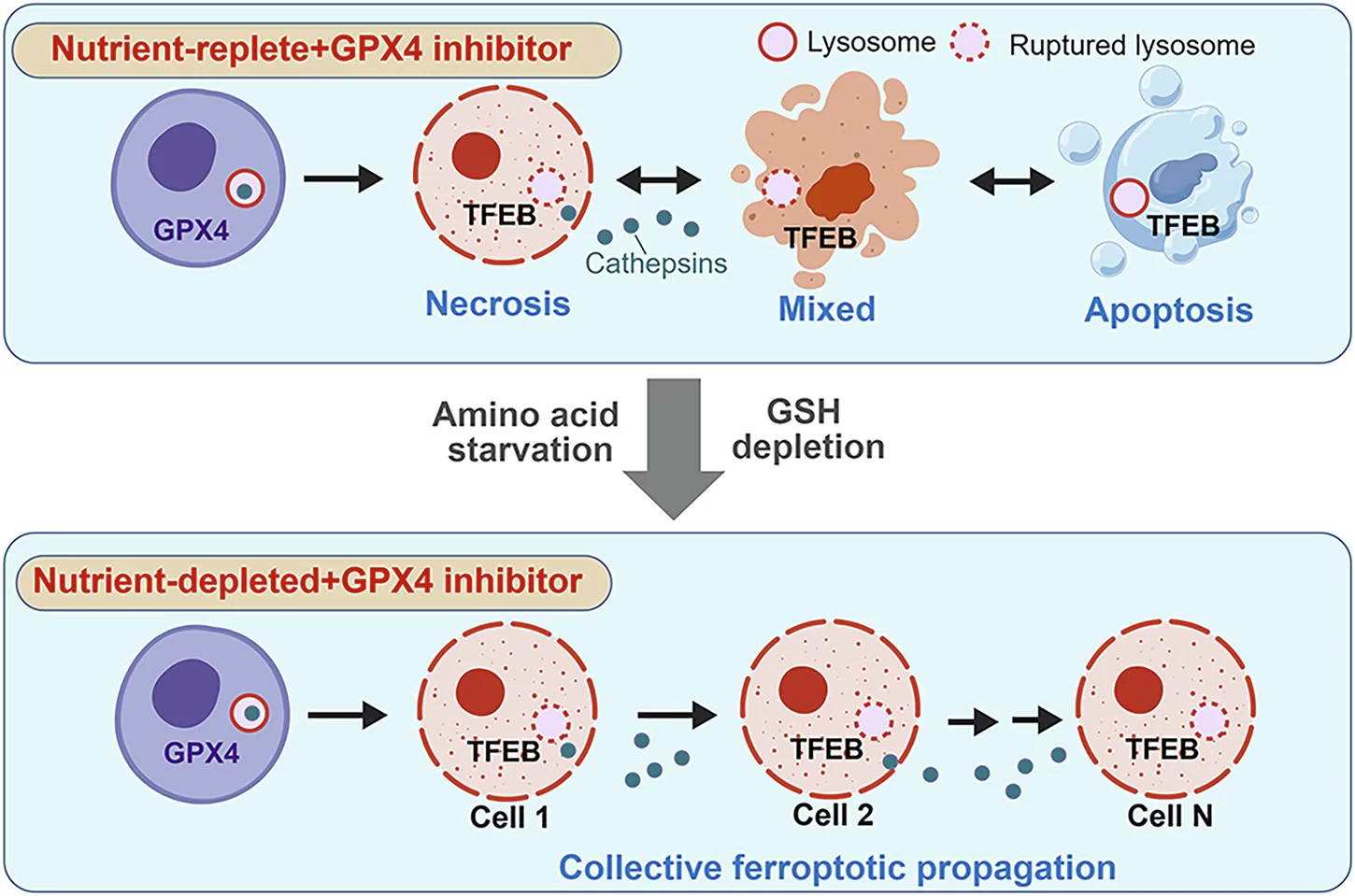
Execution heterogeneity and population-level ferroptotic propagation under distinct metabolic states. Under nutrient-replete conditions, acute GPX4 inhibition induces morphologically heterogeneous ferroptotic death, including necrotic-like, mixed, and apoptotic-like phenotypes within the same cell population. Necrotic-like cells exhibit increased lysosomal rupture and TFEB activation, which are associated with enhanced local propagation. In contrast, amino acid starvation-induced GSH depletion lowers the execution threshold, converting an initially heterogeneous, largely single-cell response into uniform, collective ferroptotic death with wave-like multicellular propagation. This figure was created with BioGDP (https://biogdp.com/).

Recent work has expanded the conceptual framework of ferroptotic propagation by demonstrating that the spread of ferroptotic injury is tightly linked to population-level heterogeneity in death execution.^89^ Under nutrient-replete conditions, acute GPX4 inhibition induces morphologically heterogeneous ferroptotic death phenotypes, in which necrotic-like, apoptotic-like, and mixed phenotypes coexist within the same cell population (Fig. 3). Spatial analyses have shown that necrotic regions display significantly greater local propagation activity than apoptotic regions, indicating that the mode of death execution itself may govern the transition from isolated cell death to multicellular ferroptotic contagion.

A key event underlying the transition toward propagative necrosis is lysosomal damage. Lipid peroxidation preferentially accumulates at lysosomal membranes and precedes lysosome rupture, transcription factor EB (TFEB) activation, and cathepsin-dependent plasma membrane breakdown, thereby positioning lysosomal stress at the center of population-scale death amplification.^89^ Under nutrient-replete conditions, these events occur in a heterogeneous manner and correlate closely with the emergence of necrotic-like vs apoptotic-like phenotypes, suggesting that lysosomal destabilization acts as a tipping point for synchronized death execution.

By contrast, under nutrient-depleted or amino acid starvation conditions, intracellular glutathione levels progressively decline, thereby lowering the execution threshold for GPX4 inhibition-induced ferroptosis. This metabolic shift converts an initially heterogeneous and largely cell-autonomous response into uniform collective necrosis accompanied by extensive lysosome rupture and wave-like multicellular propagation. In this setting, glutathione depletion acts as a critical sensitizing factor that links intracellular redox-buffering capacity to tissue-level ferroptotic spread, effectively transforming stochastic cell death into coordinated population-level contagion.

Beyond mammalian systems, the transition from single-cell ferroptosis to coordinated collective death also appears to operate in microbial populations. During the collapse of natural *Microcystis* blooms, iron-catalyzed lipid peroxidation generates bioactive truncated phospholipids that function as both executors and propagators of ferroptosis, thereby transmitting lipid peroxidation to neighboring cells and coordinating collective cell death across cyanobacterial colonies.^90^

Collectively, these findings indicate that ferroptotic propagation emerges from the interplay between intercellular transmission, the death-execution state of dying cells, and the susceptibility of recipient cells, ultimately transforming single-cell ferroptosis into coordinated population-level death programs.

## Ferroptotic propagation in development and disease

The biological relevance of ferroptotic propagation is most apparent when examined in intact developmental and disease settings.^23,91,92^ Rather than remaining a purely cell-autonomous phenomenon, ferroptosis in these contexts frequently manifests as a spatially coordinated multicellular process that resembles tissue-level death contagion.

A particularly striking physiological example comes from embryonic development. In the developing avian limb, ferroptotic cell death has been shown to propagate as millimeter-scale trigger waves during muscle remodeling, contributing to the elimination of transient embryonic muscle populations during morphogenesis.^22^ These oxidative fronts travel over long distances through tissue and display characteristics of self-sustaining propagation rather than isolated stochastic cell death. This observation is conceptually important because it argues against the view that ferroptotic spread is merely a pathological by-product of tissue injury. Instead, it suggests that controlled ferroptotic propagation may participate in normal tissue sculpting during development. However, where ferroptotic propagation begins, how far it spreads, and what ultimately terminates it remain largely unknown. Resolving these questions will be critical for establishing whether ferroptotic propagation represents a regulated morphogenetic program rather than a passive consequence of oxidative stress.

In disease, the kidney currently provides the most robust body of evidence. Beyond mechanistic models, multiple studies of ischemia-reperfusion injury, acute kidney injury, delayed graft dysfunction, and the transition from acute kidney injury to chronic kidney disease now support the presence of ferroptotic spread along renal tubular epithelia in intact disease settings, with evidence of trigger-wave dynamics, extracellular vesicle-mediated signaling, oxidized lipid mediators, and transcriptional or metabolic reprogramming circuits. In particular, coordinated tubular death and longitudinal propagation along nephron segments have been documented in both ex vivo tubular systems and in vivo murine ischemia-reperfusion models, allowing relatively strong mechanistic inference.^19^ Recent spatial multi-omics studies further support the existence of spatially coordinated ferroptosis-associated programs in injured tubules, with GPX4, ferritin heavy chain 1 (FTH1), and ferritin light chain (FTL) emerging as key spatially enriched markers in regions linked to ferroptotic spread,^93^ reinforcing the concept of ferroptosis as a spatially organized tissue response rather than an isolated cellular event.

The heart provides another important pathological context. In myocardial ischemia-reperfusion injury, ferroptosis has been implicated not only in cardiomyocyte loss and post-ischemic remodeling but also in cell-to-cell propagation of injury along aligned myofibers.^94^ Experimental studies have shown that ferrostatin-1 suppresses fiber-aligned spread of cell death following focal injury, supporting the idea that ferroptotic propagation may preferentially follow myocardial fiber orientation rather than proceed isotropically.^94^ Although the mechanistic basis remains less well defined than in the kidney, these findings suggest that highly organized tissue architecture may impose directional constraints on ferroptotic spread, effectively channeling the trajectory of injury through anatomical structure.

By contrast, evidence in neurodegeneration and inter-organ propagation remains preliminary. Proposed axes such as lung-heart ferroptotic coupling are intriguing but currently rest largely on correlative in vivo imaging, inflammatory signaling, and systemic oxidative stress markers rather than direct causal relay experiments. Distinguishing between firmly established tissue-level propagation and emerging associative models is important for maintaining interpretive balance, particularly as the field moves toward claims of systemic ferroptotic contagion.

## Therapeutic implications: amplification vs containment

The therapeutic implications of ferroptotic propagation are highly context-dependent. In ischemic and degenerative diseases, propagation represents a mechanism of collateral tissue loss and therefore should ideally be contained. Several recent studies support this view. In kidney injury models, blockade of extracellular vesicle release or suppression of bioactive lipid signaling substantially reduces the spatial expansion of tubular damage.^19,68^ Similarly, inhibition of metabolic reprogramming pathways, including lactylation-associated circuits implicated in delayed graft dysfunction, appears to blunt the propagation of ferroptotic damage across tubular epithelia.^61^ These observations suggest that limiting intercellular relay mechanisms may reduce secondary tissue loss even when the initiating insult cannot be fully prevented. Such a strategy would aim to interrupt ferroptotic contagion rather than preventing its initiation.

A particularly informative pharmacological example is provided by the diazepine derivative YL3147, a highly potent radical-trapping antioxidant that directly interrupts the chain-propagation phase of lipid peroxidation and confers robust protection in both acute and chronic murine models of doxorubicin-induced cardiomyopathy.^95^ This work provides an important proof-of-principle evidence that ferroptotic contagion may be therapeutically contained through direct interception of lipid radical amplification.

In oncology, however, the therapeutic logic may differ.^96^ In some contexts, controlled amplification of ferroptotic spread, rather than its suppression, may improve tumor clearance.^74^ Several recent studies have begun to explore this possibility. For example, nanoparticle-based lipid peroxidation amplifiers have been developed to enhance ferroptosis beyond the primary treatment zone, thereby increasing the spatial reach of therapy-induced tumor cell death.^15^ Likewise, secreted factors such as LGALS13 may enhance the susceptibility of neighboring tumor cells to ferroptosis-based treatments by lowering their antioxidant threshold,^24^ intentionally harnessing ferroptotic contagion as a therapeutic amplifier.

This creates an important therapeutic dichotomy: in normal tissues, ferroptotic propagation should generally be blocked; in tumors, it may be intentionally enhanced.

## Major unresolved controversies and current limitations

Despite the growing evidence for ferroptotic propagation, several conceptual and experimental issues remain unresolved. A central challenge is to distinguish true causal propagation from repeated exposure to a shared microenvironmental insult. In many pathological settings, neighboring cells are simultaneously exposed to hypoxia, ischemia, inflammatory mediators, iron overload, or metabolic stress.^52,54^ Under these conditions, spatial clustering of ferroptotic cells may simply reflect parallel responses to a common upstream trigger rather than directional transmission of a death signal from one cell to another. Establishing bona fide propagation therefore requires rigorous spatiotemporal evidence showing that the death of one cell directly increases the likelihood of ferroptosis in adjacent or spatially connected recipient cells.

A second unresolved issue is conceptual rather than purely experimental: whether ferroptotic propagation is best understood as transmission of discrete molecular signals or as the spatial crossing of a lipid peroxidation threshold. In this view, spread may not necessarily require a single diffusible death factor that relays lethal signals; instead, it could reflect progressive failure of local antioxidant buffering and membrane repair capacity across neighboring cells and tissue compartments. This distinction remains particularly important for interpreting trigger-wave behavior and extracellular relay mechanisms.

Another limitation is that much of the current mechanistic evidence comes from highly controlled in vitro systems, particularly two-dimensional confluent epithelial monolayers. Although these models have been invaluable for revealing trigger-wave behavior and contact-dependent spread, they may also bias interpretation by maximizing cell–cell coupling, membrane proximity, and exposure uniformity. Whether comparable propagation dynamics occur in three-dimensional tissues, where extracellular matrix constraints, variable cell density, heterogeneous antioxidant buffering, and local repair mechanisms are likely to shape spread, remains insufficiently resolved and presents a translational gap.

The specificity of current readouts also warrants caution. Lipid ROS, oxidized phospholipids, and malondialdehyde are frequently used as evidence of propagated ferroptosis, yet none of these markers is uniquely specific to ferroptotic death. Similar oxidative signatures can arise during other forms of necrotic injury, inflammatory stress, or generalized membrane damage. For this reason, propagated cell death should ideally be supported by multiple orthogonal criteria, including iron dependence, rescue by structurally distinct ferroptosis inhibitors, and preservation of canonical molecular hallmarks of ferroptosis.

Finally, direct causal evidence in vivo remains limited. Although recent work in kidney injury and developmental systems strongly supports tissue-level spread, many in vivo observations still rely on spatial correlation, endpoint histology, or ex vivo inference rather than real-time tracking of death propagation. An additional open question is whether propagating ferroptotic injury contains reversible intermediate states before reaching tissue-scale collapse or whether spread proceeds as a strictly irreversible process once initiated. Whether ferroptotic propagation represents a broadly generalizable tissue-level program or is restricted to specific biological contexts therefore remains unresolved.

## Future perspectives and concluding remarks

Taken together, current evidence supports a revised view of ferroptosis as a tissue-level death program that can extend beyond the initiating cell. Rather than acting solely as a cell-autonomous execution pathway, ferroptosis may, under permissive conditions, propagate across tissues through interconnected modes that include redox trigger waves, membrane-contact transfer, and extracellular paracrine signaling, collectively defining the multiscale architecture of ferroptotic contagion.

A key challenge for the field is to integrate these apparently distinct mechanisms into a unified multiscale and context-dependent framework. A central question is whether these modes represent fundamentally different biological processes or instead reflect distinct spatial regimes of the same propagative program. One possible unifying model is that direct membrane transfer predominates in short-range spread, whereas redox trigger waves and extracellular relay mechanisms govern propagation over medium to long distances, thereby establishing a hierarchy of spatial coupling mechanisms. These spatial regimes are not necessarily mutually exclusive. Large-scale ferroptotic spread may emerge from the cumulative effect of repeated local transmission events, whereby oxidative stress is regenerated from one cell to the next. In this view, the distinction between short- and long-range propagation reflects differences in the dominant mode of coupling and spatial scale rather than fundamentally separate biological processes. Determining whether these regimes are mechanistically distinct or simply represent different scales of the same propagative process remains an important challenge for future investigation. Another important question is whether distinct propagation modes have different metabolic and immunological signatures, leading to divergent biological outcomes.^97,98^

Equally important is to define the boundaries of spread. Tissue geometry, extracellular barriers, stromal interactions, and local antioxidant niches are all likely to shape whether ferroptotic injury remains spatially restricted or progresses to tissue-scale death programs. Whether ferroptotic propagation can extend beyond local tissue architecture to mediate true inter-organ communication remains unresolved. Recent lung–heart axis studies provide an intriguing framework for this possibility, but causal evidence for long-range cell death propagation across organs is still lacking.^99^

Another major challenge is selectivity. In ischemic and degenerative diseases, ferroptotic spread is likely to be pathogenic and should be contained. In contrast, controlled amplification of propagation may be therapeutically advantageous in tumors. Progress in both directions will depend on spatially resolved biomarkers, tissue-targeted delivery strategies, and quantitative models capable of predicting propagation thresholds and wave dynamics.

A clearer understanding of how ferroptotic spread is initiated, constrained, and manipulated may ultimately reposition ferroptosis from a cell death mechanism to a broader principle of tissue-level regulation. A key conceptual priority for the field will be to distinguish direct signal transmission from the propagation of ferroptosis susceptibility states, as these processes may reflect fundamentally different modes of tissue-level coordination.
